# Study on micromolecular mechanical properties of C-atom reinforced SBR polymer composites

**DOI:** 10.1038/s41598-023-49640-x

**Published:** 2024-01-02

**Authors:** Hongyue Chen, Siyuan Liu, Jingdao Fan, Pengfei Li

**Affiliations:** 1 School of Mechanical Engineering, Liaoning Technical University, Fuxin, 123000 China; 2 Department of Shanxi Yanchang Petroleum (Group) Co., Ltd., Xi’an, 710075 China

**Keywords:** Mechanical engineering, Engineering, Materials science, Theory and computation

## Abstract

A three-layer microscopic model with Fe atoms as the top and bottom layer and SBR polymer composites as the middle layer and SBR polymer composite was established and studied. By adding C atoms as reinforcement, the stability and elastic modulus and frictional coefficient changes of SBR polymer composites before and after adding C atoms were studied. In this study, the molecular dynamics method was used to change of elastic modulus was observed by stretching, compression and shear of the SBR polymer composite; The simulation shows that after adding C atom the elastic modulus of SBR polymer composite increased, the friction coefficient of polymer composite upper and lower decreases and the relative atomsic concentration, temperature, velocity, overall temperature average, kinetic energy, total energy and MSD in the thickness direction are reduced after adding C atoms. The stability of SBR polymer composites is enhanced, and the deformation under shear is weakened. In addition, it is found that the binding energy between SBR polymer composites and Fe atoms is reduced after adding C atoms.The stability of SBR polymer composites is improved during use. This work provides a method for studying the properties of rubber composites by studying the enhancement of the stability of SBR polymer composites from the microscopic point of view.

## Introduction

Styrene butadiene rubber is a kind of rubber commonly used in conveyor belt fields. This paper takes it as the research object. The elastic modulus, frictional coefficient, and material stability of rubber in actual use is an essential factor affecting the operational reliability of conveyor belts. The steel wire rope core is used as the skeleton of the rubber conveyor belt, and rubber is used as the outer covering layer. It is necessary to study the interaction characteristic between Fe atoms and SBR polymer composites from the microscopic point of view, and mechanical properties of the SBR polymer composites. With the industry's development, rubber's performance has been challenged more. In the current research on rubber materials, silica, graphene, and other materials are used as reinforcing agents of rubber materials to improve rubber materials' performance and mechanical properties, processing properties, and tensile strength of rubber.

Some experts have studied the properties of rubber materials reinforced by filler. Li et al.^1^ studied the interaction between the polymer matrix and the graphene-reinforced interface using molecular dynamics and tensile simulation. The molecular layer model with Fe atoms as the top nanolayer is established and the tribological properties of the polymer composites are enhanced by sliding the top Fe nanolayer on the surface of the polymer matrix. More polymer chains are readily adsorbed and bonded on the surface of the HOFG-reinforced body, resulting in less interaction of the polymer chain with the top metal layer. Therefore, better tribological properties (i.e., lower friction coefficient and wear rate) can be obtained. In Li et al.^2^, iron nanorods were used to slip on the top of polymer reinforced with carbon nanotubes and polymer reinforced without carbon nanotubes at different speeds to analyze the influence of carbon nanotubes on the friction coefficient and wear rate of the polymer at different speeds. Molecular models were developed with iron atoms as top nanorod and bottom layers and with polymer/carbon nanotube composite as the core, showing that incorporating carbon nanotubes reduced the composite's average friction coefficient and wear rate by approximately 22% and 21%, respectively. In Li et al.^3^, molecular models of the original, functionalized, and cross-linked graphene sheet/polymer composites used the temperature cooling method to study the increase of glass transition temperature of materials before and after adding graphene sheet reinforcement. Compared with the original graphene sheet/polymer composite, the glass transition temperature of cross-linked graphene sheet/polymer composite is increased by about 12.2% and 8.9%, respectively. Computing the interface interaction energy, the radius distribution function matrix between GS and polymer, the Mean Square Displacement value, and the free volume of polymer composite, it can be found that covalent bonds play an essential role and can improve the thermal properties of the whole polymer composite. In Yang et al.^4^, a molecular model of the expanded nitrile rubber (NBR) matrix enhanced by both carbon nanotubes (CNT) and graphene (GN) is established, the tensile and shear behaviors of rubber composites reinforced with carbon nanotubes and graphene were studied by molecular dynamics method under the expanded state. The introduction of CNT and GN significantly improved the mechanical properties of the expanded NBR matrix. Compared with carbon nanotubes, the elastic modulus is about 24.95%, tensile strength is about 7.54%, and shear modulus is about 5.96%. In Li et al.^5^ developed a molecular model of the graphene-enhanced polymer composite and studies enhancing the mechanical and tribological properties of the composite by introducing graphene as an enhancement; graphene was used to strengthen the polymer, and the constant strain method was used to discuss the changes of Young's modulus and shear modulus before and after polymer reinforcement. The addition of graphene sheets increases Young's modulus of the polymer composite by about 150% and the shear modulus by 27.6%. The tribological and kinematic characteristics of rubber under the action of external force were discussed in the above study.

At the current stage, molecular dynamics (MD) simulation has been widely used in molecular simulation. Various simulation studies are reviewed on modeling, calculating, and analyzing enhanced elasticity, tensile, and fracture properties of carbon nanotubes and graphene sheet/polymer composites. Shi et al.^6^ studied the thermodynamic properties of SBR through molecular dynamics (MD) simulation combined with experimental methods. Studies have shown that lower styrene content makes SBR have better thermal stability, and the inclusion of styrene/vinyl limits the movement of polymer chains. Fu et al.^7^ studied the mechanical properties of SBR before and after adding PPA. By combining molecular dynamics (MD) simulation and experiment, it was found that the elastic modulus and stiffness of the material were improved and the creep recovery ability was enhanced after adding PPA. Luo et al.^8,9^ studied the effect of BTC on the vulcanization process and anti-oxidation of SBR and the anti-oxidation process of MBC in SBR by molecular dynamics (MD) simulation combined with experiments. The results showed that the addition of BTC could accelerate the vulcanization reaction and weaken the negative effect of vulcanization reaction. The addition of MBC could significantly reduce the effect of SBR crosslinking density in the oxidation process. Joseph et al.^10^ studied the effect of NP on the strength of SBR. Molecular dynamics (MD) simulation method was used to determine which factors had a significant effect on the strength and dispersion of NP by monitoring the peak normal traction, strength modulus, interface energy, rotation radius, distance between NP and other parameters of different systems. The results show that NP can enhance the strength of SBR, and the strength of SBR increases with the increase of NP volume fraction and particle size. Guo et al.^11^ used MD simulation method to simulate the friction process between tire rubber and aggregate. After simulation, it was found that the friction coefficient of SBR was greatly affected by temperature, and the change of friction coefficient with temperature was mainly affected by the average vertical stress. Zhou et al.^12^ studied the relationship between SiO_2_/SBR interface structure and mechanical properties by molecular dynamics (MD) simulation. The results show that KH550 with different grafting fractions will affect the intermolecular and intramolecular interactions of SBR/silica, thus affecting the number of adsorption chains on the surface of the filler, the number and length of strings, rings and tails, the flexibility of the body and the mechanical behavior of interface deformation. Li et al.^13^ discuss the mechanical and tribological properties of polymer composites reinforced by graphene sheets and carbon nanotubes through molecular dynamics simulation from an atomic perspective. Li et al.^14^, a three-layer molecular model with Fe atoms as the top and bottom layer and the polymer and carbon nanotube matrix as the core was established and studied to improve the tribological properties of polymer composites by using carbon nanotubes as a reinforcing material, discussed the influence of carbon nanotubes on the tribological properties of polymer composites by shear simulation. The simulation results show that the shear modulus of the composite can be increased by up to 60% by introducing carbon nanotubes. In Li et al.^15^, the polymer matrix of carbon nanotubes and graphene sheets was built, and carbon nanotubes and graphene were used to enhance the polymer separately. The constant strain method was used to discuss the interaction between the surface of the reinforcement material and the polymer matrix material in the process of pulling out carbon nanotubes and graphene and to analyze the mechanical properties of the polymer composite. The results show that the composite has an 18% higher Young modulus, 8.7% higher tensile strength, and 5% higher surface crack energy than carbon nanotubes. In Li et al.^16^, we developed a molecular cross-linking model of a pure epoxy matrix with an initial single-blade crack, tensile strength, and elongation at the break of carbon nanotubes before and after reinforcement were studied by constant strain method. The results showed that the tensile strength and fracture elongation of CNT/epoxy composites increased by 24.8% and 34.3%, respectively. In He et al.^17^, building a molecular model of nano silica particle-enhanced polymer composites, the tribological properties of polymer composites before and after the addition of nano-sio2 particles were studied utilizing molecular dynamics simulation and the sliding method of the top iron layer. We showed that Young's modulus increased by about 190% after introducing nano-silica particles. The average friction coefficient and wear rate of the polymer/nano-silica composite were reduced by approximately 27% and 47.4%, respectively. To explore the mechanism of the tribological performance enhancement, we calculate and discuss the interface interaction between the polymer material and the nanometer silica particles, the radius distribution function value between the top iron layer and the polymer material, and the atomic concentration of the polymer composites. In Li et al.^18^ developed molecular models with iron atoms as the top nanorod and bottom layer, polymer/carbon nanotube composite as the core, and shear simulation was used to discuss the influence of NBR polymer on tribological properties before and after the addition of carbon nanotubes. The simulation results show that the average friction coefficient of the composite is reduced by 38%, and the average wear rate decreases by 60% by introducing carbon nanotubes.

Moreover, the friction coefficient and wear rate undergo two phases during loading. The first stage is a stable fluctuation period, and the second stage is a significant increase in the coefficient and wear rate. In Guan et al.^19^, the RMD simulation based on the reaction force field compared the initial decomposition temperature, the distribution of intermediates and products, and the related kinetic characteristics of the pyrolysis process of nitroethane and methylcyclohexane (MCH). All the above studies indicate that it is feasible to use MD simulation to study molecular dynamics.

Chaudhry et al.^20^ prepared graphene oxide to study the effect of adding different fillers on the polymer properties by tensile experiments. Cheng et al.^21^ studied the polyethylene (PE) in reducing graphene oxide (RGO) nanosheet of epitaxial crystallization using a polarized light microscope, scanning electron microscope, transmission electron microscope, and atomic force microscope studied the RGO-induced PE crystal morphology in the presence of 2-dimensional RGO nanosheet, the crystallization dynamics of pgn is significantly accelerated, in the presence of a small amount of RGO nanosheet, the thermal stability of PE has significantly improved. Muhammad et al.^22^ studied a method of mixing PE and oxidized PE (OPE) to improve graphene dispersion in polyethylene (PE), simulated the mechanical properties of nanocomposites, studied the microstructure of nanocomposite by small angle x-ray scattering, confirmed the graphene dispersion, graphene formed surface fractality in the composite. Saibom et al.^23^ studied the influence of the dispersion degree of nanoparticles on the properties of polymer nanocomposites. We studied the tensile modulus of rubber with different dispersion degrees in PE and discussed the influence of polymer nanocomposites with different dispersion degrees on the tensile modulus of rubber. Shen et al.^24^, using silica acid as a silica precursor, polyamide-6/silica nanocomposite, add coupling agent (γ -aminopropyl) triethoxysilane, introduced the interface interaction between silica and polymer matrix, discuss the influence on the material performance, the results show that the inorganic component incorporation significantly improves the melt viscosity, tensile strength, Yang’s modulus, thermal decomposition temperature, glass transition temperature and polyamide-6 softening temperature of the resin. However, for the enhancement of the properties of rubber composites by adding fillers, it is costly and time-consuming to adopt the macro research method, and it cannot reveal the changes in the molecular stability of rubber composites after adding fillers at the micro level. Therefore, it is necessary to carry out the research at the atomic level.

The existing research studies on the stability of rubber polymer composites are relatively few. Liu et al.^25^ point out that the random distribution of reinforcement materials can improve rubber polymer composites' reinforcement effect. Common reinforcement materials include carbon black, carbon nanotubes, and graphene sheets, whose main components are carbon. Since carbon atoms can be randomly distributed to the maximum extent, carbon atoms are randomly distributed in rubber polymer composites in this paper, and the changes in the stability of rubber polymer composites before and after reinforcement with carbon atoms are analyzed. Therefore, through the method of molecular dynamics simulation, The Elastic modulus, Frictional coefficient, Velocity, Relative concentration, Temperature, Overall temperature average, Kinetic energy and Total energy, mean square displacement (MSD), and Cohesive energy of rubber polymer composites were studied under different temperature conditions. Changes in strength and stability of rubber polymer composite before and after addition of carbon atoms are discussed.

## Model

**Figure 1:**
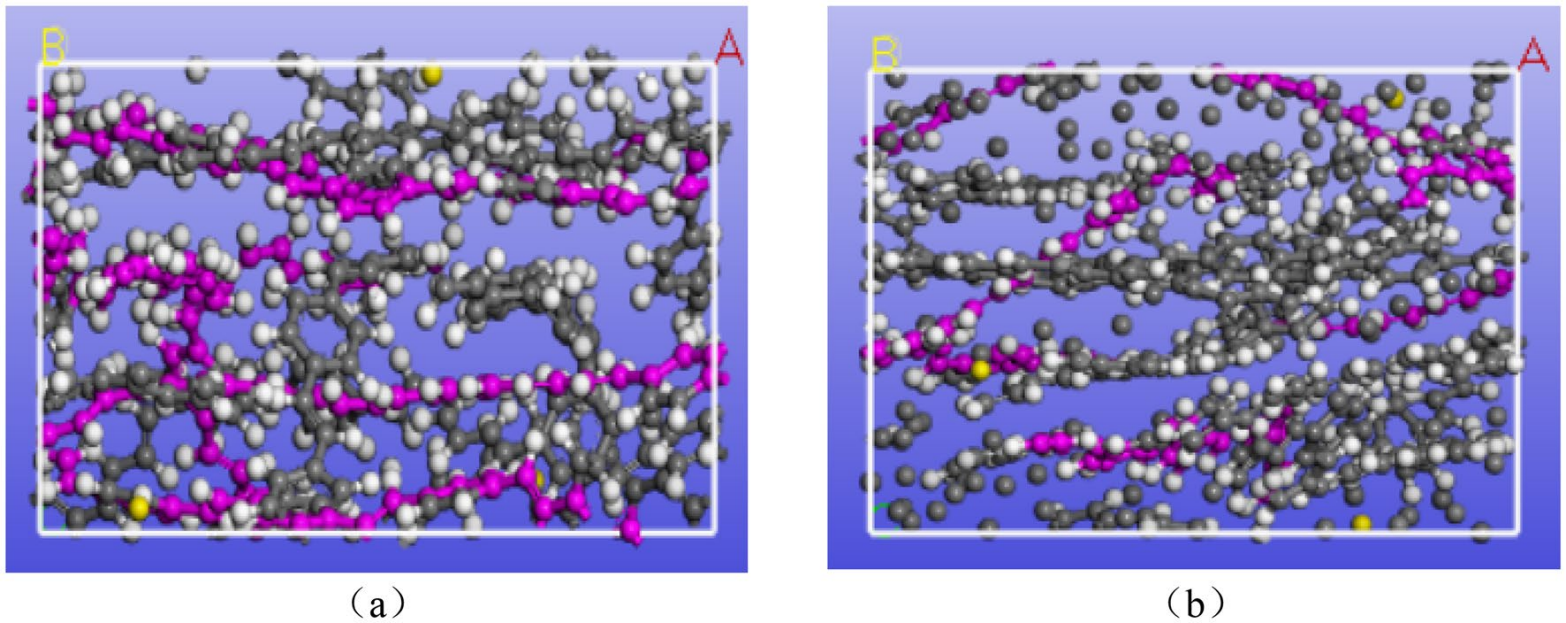
SBR polymer model: (**a**) before the addition of carbon atoms; (**b**) after the addition of carbon atoms.

In order to study the influence of adding carbon atoms on the effect of the strength and the stability of SBR polymer composites, Material Studio software was first used to establish two models, (a) and (b), as shown in Fig. 1. Both models are SBR polymer composites, in Fig. 1, the upper and lower layers are both Fe layers, and the middle layer is SBR polymer composite material. As repeating units, SBR is formed by copolymerizing 1,3 butadiene (CH2=CH–CH=CH2) and styrene (C6H5–CH=CH2). The Forcite module in Material Studio is adopted to simulate the interaction between molecules, and the COMPASS II force field is adopted. Using COMPASS II force field, COMPASS II force field is the extension of COMPASS force field, supports condensed matter material atomic level simulation of powerful force field, is parametric and verified the ab initio force field, and added a variety of isolated system simulation with ab initio and empirical data. Therefore, the force field can accurately and simultaneously predict the structure, conformation, vibration, and thermodynamic properties of many isolated and condensed molecules in a wide range of temperatures and pressure and can be used to describe the mechanical properties of polymers.

Under COMPASS II force field, the total potential energy E can be summarized as formula (1):

1 $$\begin{aligned} E & = \sum {E^{(b)} } + \sum {E^{(\theta )} } + \sum {E^{(\gamma )} } + \sum {E^{(\Phi )} } + \sum {E^{{(bb^{{\prime }} )}} } + \sum {E^{(b\theta )} } + \sum {E^{(b\Phi )} } + \sum {E^{{(b^{{\prime }} \Phi )}} } \\ & \quad + \sum {E^{{(\theta \theta^{{\prime }} )}} } + \sum {E^{(\theta \Phi )} } + \sum {E^{{(\theta \theta^{{\prime }} \Phi )}} } + \sum {E^{(vdw)} } + \sum {E^{(elec)} } \\ \end{aligned}$$

**Figure 2:**
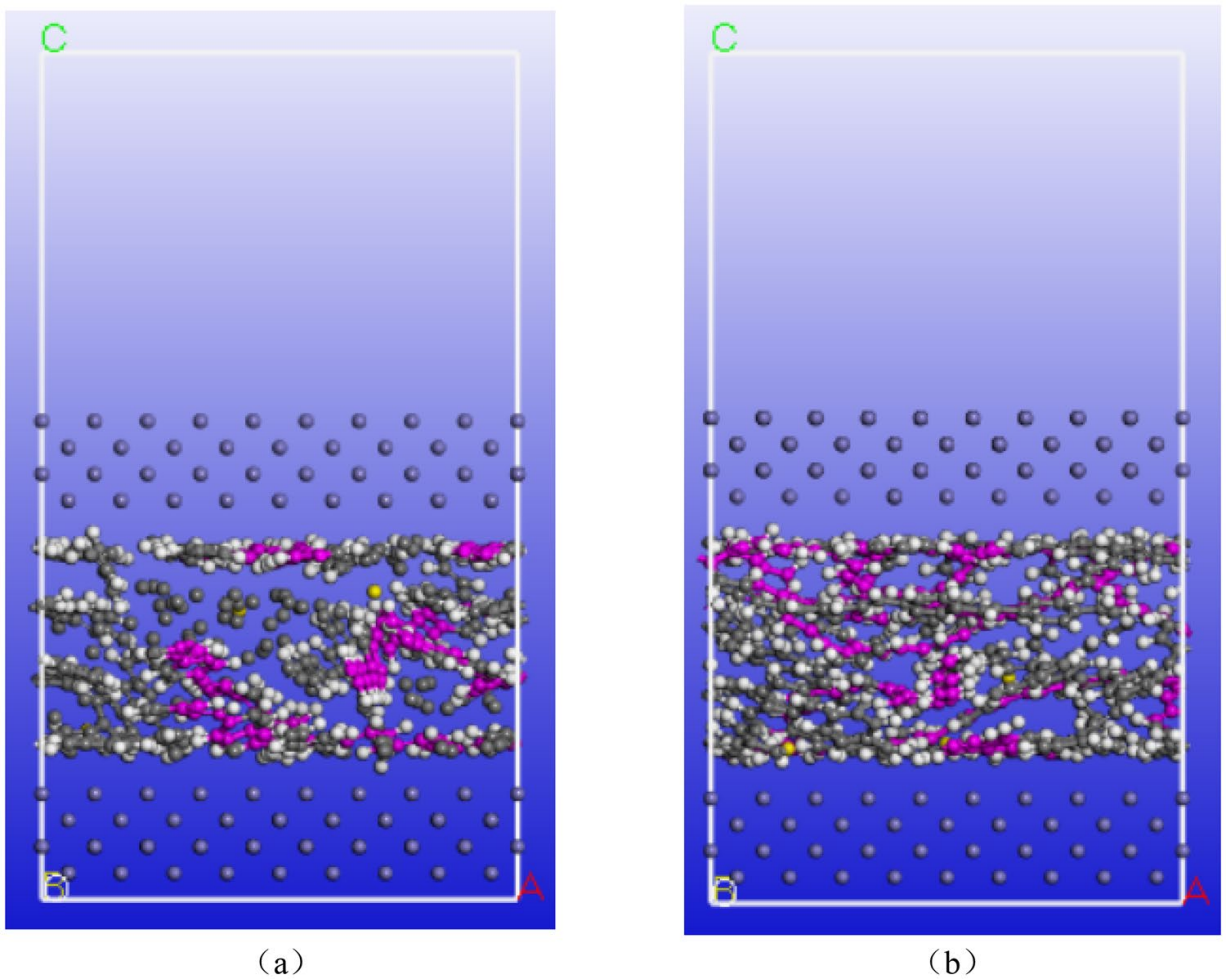
A three-layer model: (**a**) before adding carbon atoms; (**b**) after adding carbon atoms.

The b and b ′ are the bond lengths between two adjacent atoms, the $\theta$ and $\theta^{{\prime }}$ are the adjacent double bond angles, $\Phi$ is the dihedral torsion angle, $\gamma$ is the outer plane angle, the potential energy can be divided into two categories, one is the internal valence term namely ($E^{(b)} ,E^{(\theta )} ,E^{(\Phi )} ,E^{(\gamma )}$), and the cross-coupling term namely ($E^{{(bb^{{\prime }} )}} ,E^{(b\theta )} ,E^{(b\Phi )} ,E^{{(b^{{\prime }} \Phi )}} ,E^{{(\theta \theta^{{\prime }} )}} ,E^{(\theta \Phi )} ,E^{{(\theta \theta^{{\prime }} \Phi )}}$). Meanwhile, $E^{(vdw)}$ is reflected by the sum of repulsive force and attraction. In addition, the $E^{(elec)}$ non-bonded interactions up to the radius are selected at 1.25 nm. The SBR polymer uses three polymer chains, each containing 20 repeat units, and is made of butadiene (CH_2_=CH–CH=CH_2_) and styrene (C_6_H_5_–CH=CH_2_) in the target ratio of 10:10. The same three molecular chains are used in the polymer reinforced with carbon atoms. Carbon atoms are added and then polymerized. In the modeling process, the Conjugate gradient method will be used to minimize the energy, and the convergence accuracy will be 0.005 kcal/mol/Å reasonable model will be established. Then, Anneal module will be used to perform annealing under the condition of constant temperature and constant volume ensemble (NVT). The annealing temperature is 323–473 K, and the cycle is 5 times, each cycle is heated 50 times, and a total of 50,000 times of equilibrium is passed to further relax and reduce the interaction force between molecules. Then, the “Quench” module is used for cooling, and the temperature is reduced to 298 K, after 5000 steps, and the convergence accuracy is 0.005 kcal/mol/Å. In Fig. 2, both the upper and lower layers are Fe layers, and the middle layer is SBR polymer composite material established in Fig. 1.

**Figure 3:**
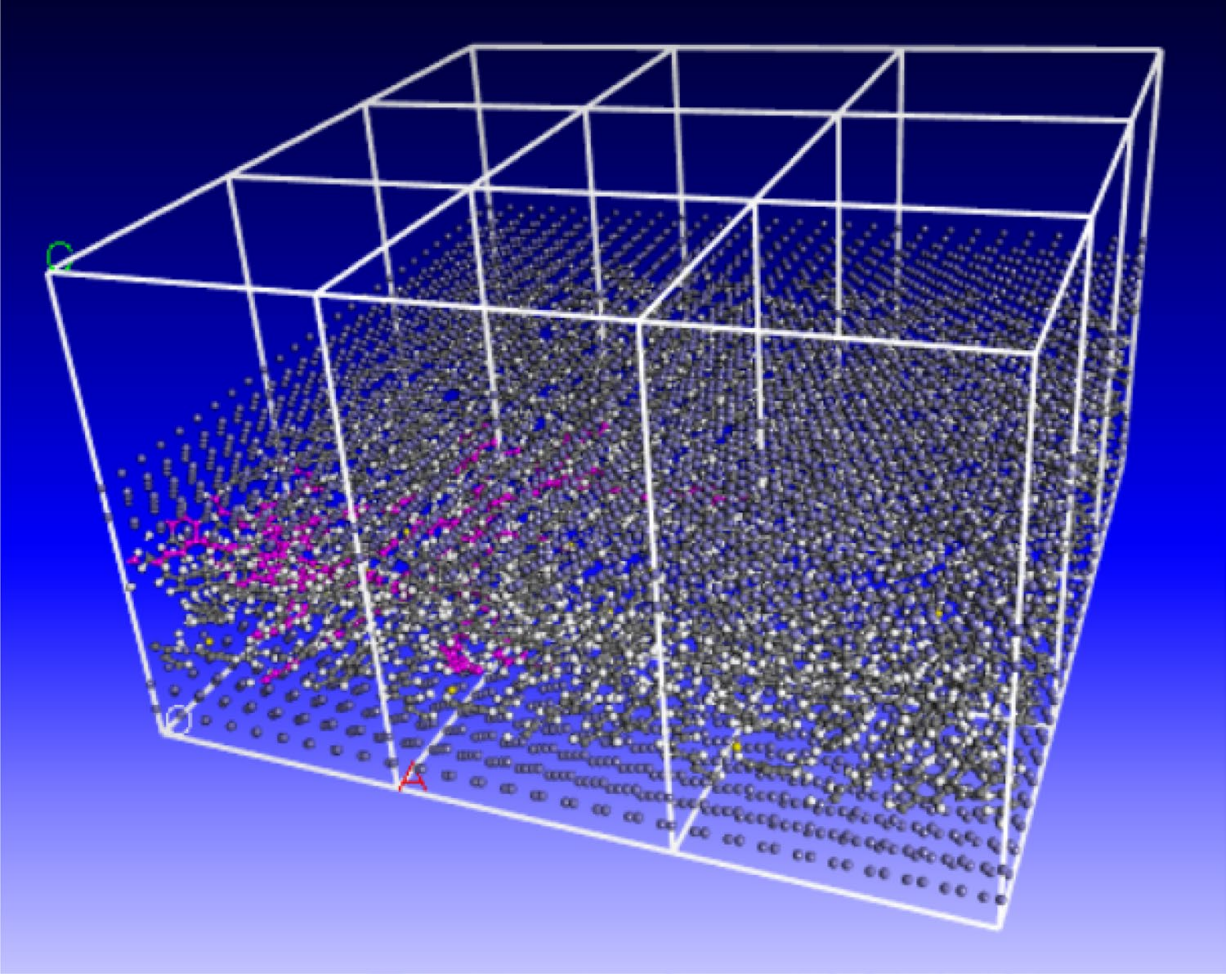
A Schematic diagram of the periodic structure.

As the steel wire rope is the skeleton material of the rubber conveyor belt and its main component is Fe, there is an interaction between the steel wire rope skeleton and the rubber matrix of the conveyor belt. Therefore, the upper and lower parts of the model are set as Fe atoms, with a size of 2.5 × 2.5 × 0.57 nm^3^. Next, the pure SBR polymer used in this study and the SBR polymer composite material with carbon atoms were constructed, respectively. Then it uses the “Build Layer” module to establish a three-layer model of the upper and lower layers as Fe atoms and the central position of SBR polymer composites. The SBR polymers of the middle layermass density was 1.04 g/cm^3^, and the boundary conditions between the middle-layer SBR polymer and the three-layer model are all periodic boundary conditions. As shown in Fig. 3, periodic boundary conditions, crystal cell in a three-dimensional direction along the infinite extension, is also the boundary atoms repeat, not entirely independent unit, can be seen as a structure of repeated accumulation, in addition, z direction is periodic, but because the vacuum layer, so multiple crystal cells will not affect each other.

Before the shear simulation, the structure was optimized in the “Geometry Optimization” module, and the convergence accuracy was set at 0.005 kcal/mol/Å to complete the geometric optimization. Next, annealing is performed under constant temperature and volume ensemble (NVT). The Annual module is used to complete the annealing process. The annealing temperature is from 323 to 473 K, and the annealing cycle is performed five times, with each cycle heated 50 times. Then, the "Quench" module is used for cooling, and the temperature is reduced to 298 K; after 5000 steps, the convergence accuracy is 0.005 kcal/mol/Å. Confined to the Confined Shear module, Fe atoms are confined to the confined shear module with a movement speed of 0.01 Å /ps (1 m/s) and a simulation time of 1500 ps. The polymer composite material in the center is subjected to shear simulation. Fe atoms on the upper and lower layers move at the same speed but in opposite directions.

In the following simulation, analyzing the elastic modulus and shear elastic modulus of stretching and compression of SBR polymer composite materials, studying the strength changes of SBR polymer composite materials before and after the addition of C atoms, and changes of the sliding friction coefficient between Upper and Lower to analyze the degree of interaction between Fe atoms and SBR polymer composites, Friction coefficient of Upper and Lower under shear, velocity, temperature, and relative atomic concentration in the thickness direction under the shear action of the three-layer structure were used to judge the strength of the current atomic interaction between polymers by the velocity, the energy dissipation by the temperature and the atomic motion by the relative atomic concentration, the friction coefficient determines the energy accumulation between the Fe atomic layer and the SBR polymer composite. It also analyzes the temperature, kinetic energy, total energy, and pressure of the whole system in the simulation process to judge the energy characteristics of polymer composites under shear load and the current stability of polymer composites through energy analysis.

## Results and discussion

### Isothermal condition

#### Elastic modulus

The most common loads in the actual work of materials are tensile load, compression load and shear load. The Dynamics module of Material Studio is used to load tensile load, compression load and shear load respectively on SBR polymer composite materials, and the load starts from 0.1 Gpa. Each increase of 0.1 Gpa uniformly increased to 1.5 Gpa, and the change of the strength of SBR polymer composite after adding C atom was studied through the change of elastic modulus of SBR polymer composite under different loads. Since temperature can affect the mechanical properties of SBR polymer composites, it is necessary to load them at different temperatures of − 40 °C, − 30 °C, − 20 °C, − 10 °C, 0 °C, 10 °C, 20 °C, 30 °C, and 40 °C respectively. Due to the similar effect of temperature on elastic modulus, due to space limitation. The characteristics of elastic modulus at each temperature are not introduced. In most cases, rubber material equipment is used at room temperature, because there is a certain friction during use, the temperature of rubber material is often around 30 °C. Therefore, 30 °C is selected for load loading.

In this paper, sulfurization treatment of SBR material and sulfurization treatment of SBR material after adding C atom enhancement were selected respectively. The materials of sulfurization treatment of SBR material were mainly SBR and S, which were represented by SBR-S in this paper. After adding C atom to the SBR material, the vulcanization treatment mainly consists of SBR, S and C, which is represented by SBR-S-C in this paper. The changes of elastic modulus of SBR polymer composites under tensile, compressive and shear loads are discussed.

**Figure 4:**
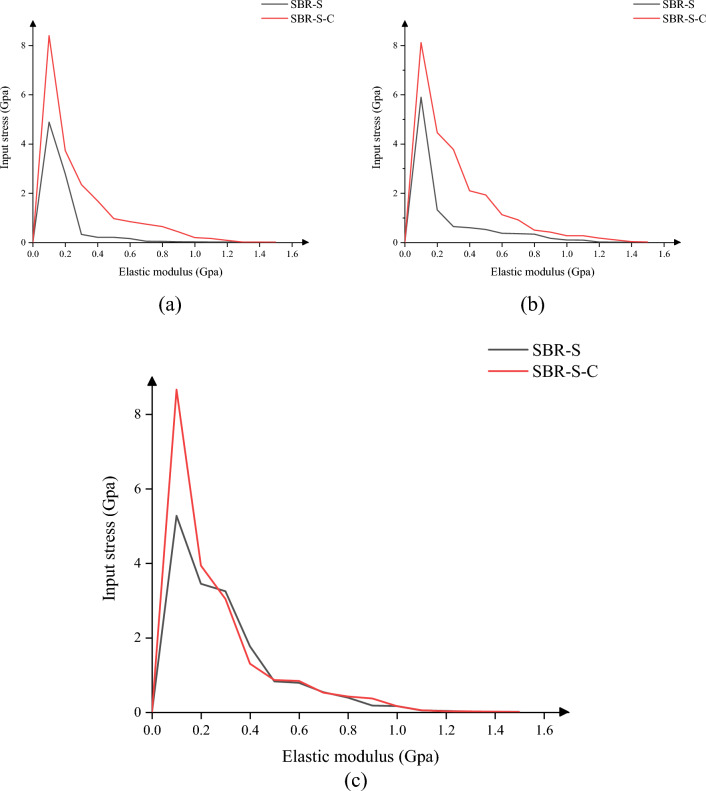
Elastic modulus under tensile load: (**a**) elastic modulus under X direction; (**b**) elastic modulus under Y direction; (**c**) elastic modulus under tensile load in Z direction.

Since the most common load borne by the material is the tensile load, the tensile load is first used for loading. As shown in Fig. 4, the tensile elastic modulus of the SBR polymer composite reinforced with C atom increased in X, Y and Z directions. The maximum elastic modulus in X direction increased from 4.89 to 8.41 Gpa, an increase of 72.02%, and the average elastic modulus increased from 0.58 to 1.35 Gpa. 132.43% increase. The maximum elastic modulus in the y-direction was increased from 5.90 to 8.12 Gpa by 37.67%, and the average elastic modulus was increased from 0.69 to 1.61 Gpa by 132.94%. The maximum elastic modulus in the Z direction was increased by 64.31% from 5.27 to 8.67 Gpa, and the average elastic modulus was increased by 21.07% from 1.11 to 1.34 Gpa. It can be concluded that adding C atom to SBR polymer composites can effectively improve the elastic modulus of polymer composites and increase the elastic modulus of polymer composites under tensile action. Under the same load size, the deformation of polymer composites will be reduced, the interaction between atoms will be closer, and the tensile strength of materials will be significantly improved.

**Figure 5:**
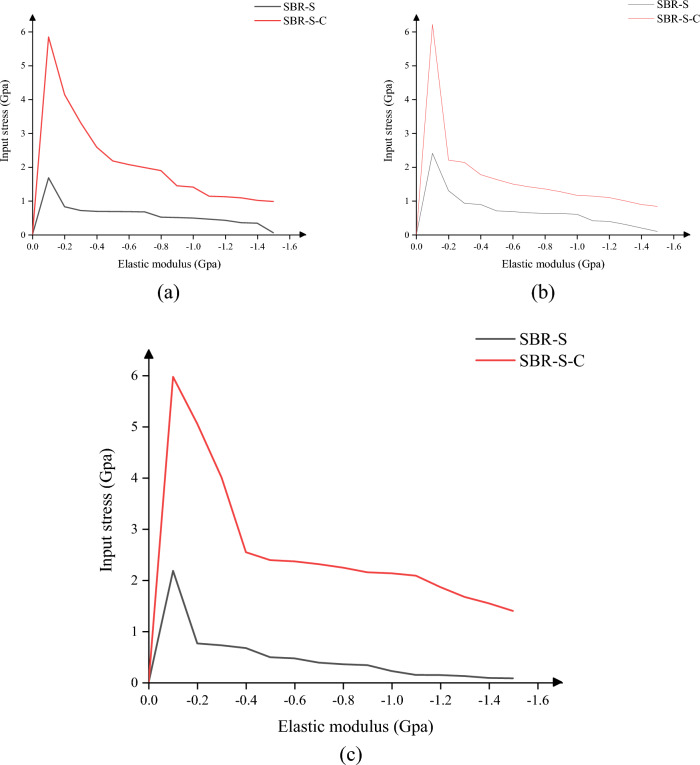
Elastic modulus under compressive load: (**a**) elastic modulus under compressive load in X direction; (**b**) elastic modulus under compressive load in Y direction; (**c**) elastic modulus under compressive load in Z direction.

In this paper, we discuss the change of elastic modulus of SBR polymer composites under the conditions of compression loading. Since the direction of the force is opposite to the coordinate direction when the compressive load is applied to the material, the value of the force is negative. As shown in Fig. 5, the SBR polymer composite was strengthened after the addition of C atom, and the compressive elastic modulus in X, Y and Z directions were improved. The maximum elastic modulus in X direction was increased from 1.68 to 5.85 Gpa, an increase of 248.33%, and the average elastic modulus was increased from 0.60 to 2.15 Gpa. 255.81% improvement. The maximum elastic modulus in the y-direction was increased from 2.40 to 6.22 Gpa by 158.65%, and the average elastic modulus was increased from 0.72 to 1.71 Gpa by 137.64%. The maximum elastic modulus in the Z direction was increased by 174.24% from 2.18 to 5.97 Gpa, and the average elastic modulus was increased by 458.89% from 0.47 to 2.65 Gpa. It can be concluded that adding C atom to SBR polymer composite can effectively improve the elastic modulus of polymer composite and increase the elastic modulus of polymer composite under compressive load. The deformation of polymer composite under the same load will be reduced, the interaction between atoms will be closer, and the compressive strength of the material will be significantly improved.

**Figure 6:**
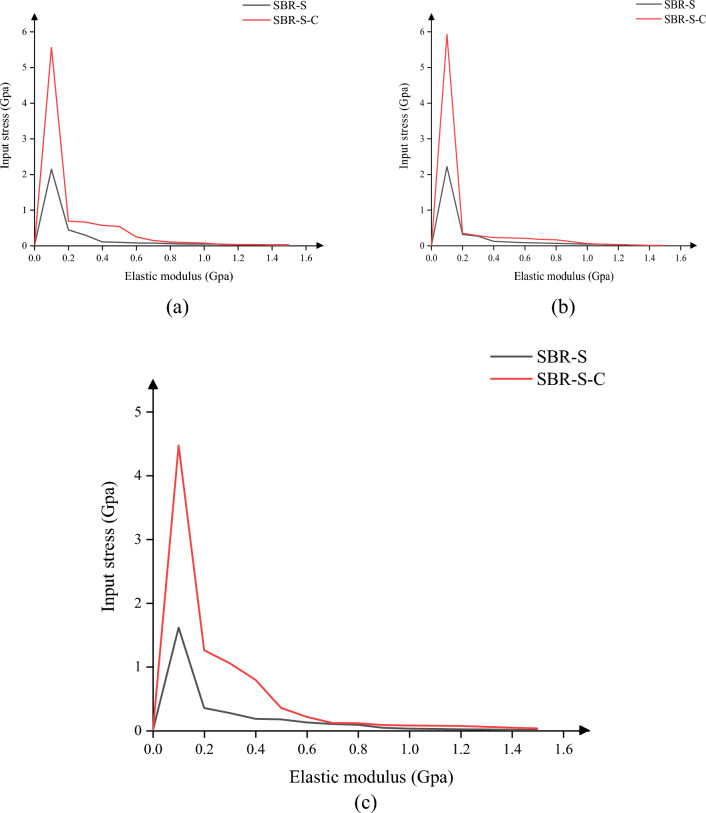
Elastic modulus under shear load: (**a**) elastic modulus under XY plane shear load; (**b**) elastic modulus under XZ plane shear load; (**c**) elastic modulus under YZ plane shear load.

The material is not only subjected to tensile and compressive loads, but also to shear loads. As shown in Fig. 6, the SBR polymer composite is strengthened after the addition of C atom, and the compressive elastic modulus in the XY plane, XZ plane and YZ plane is improved, among which the maximum elastic modulus in the XY plane is increased from 2.15 to 5.56 Gpa. The average elastic modulus increased by 159.28% from 0.23 to 0.58 Gpa, an increase of 152.53%; The maximum elastic modulus of the XZ plane is increased from 2.22 to 5.93 Gpa, an increase of 167.57%, and the average elastic modulus is increased from 0.22 to 0.52 Gpa, an increase of 130.44%. The maximum elastic modulus of YZ plane is increased from 1.61 to 4,48 Gpa by 177.61%, and the average elastic modulus is increased from 0.20 to 0.59 Gpa by 193.03%. It can be concluded that adding C atom to SBR polymer composite can effectively improve the elastic modulus of polymer composite under shear load, and the deformation of polymer composite under the same load will be reduced, the interaction between atoms will be closer, and the shear resistance of the material will be significantly improved.

In summary, the addition of C atom to SBR polymer composites can effectively improve the elastic modulus of SBR polymer composites, and the elastic modulus of SBR polymer composites under tensile and compressive loads in X, Y and Z directions is improved. When shear loads are applied to the XY, XZ and YZ planes, the elastic modulus of SBR polymer composites is improved. The shear modulus of SBR polymer composite was also significantly improved. It can be inferred that the rubber conveyor belt material in the case of uniform distribution of atoms can improve the tensile pressure and shear resistance of the material, the mechanical properties of the material have been significantly improved, and the actual work of the rubber conveyor belt is more stable.

#### Kinematic simulation

**Figure 7:**
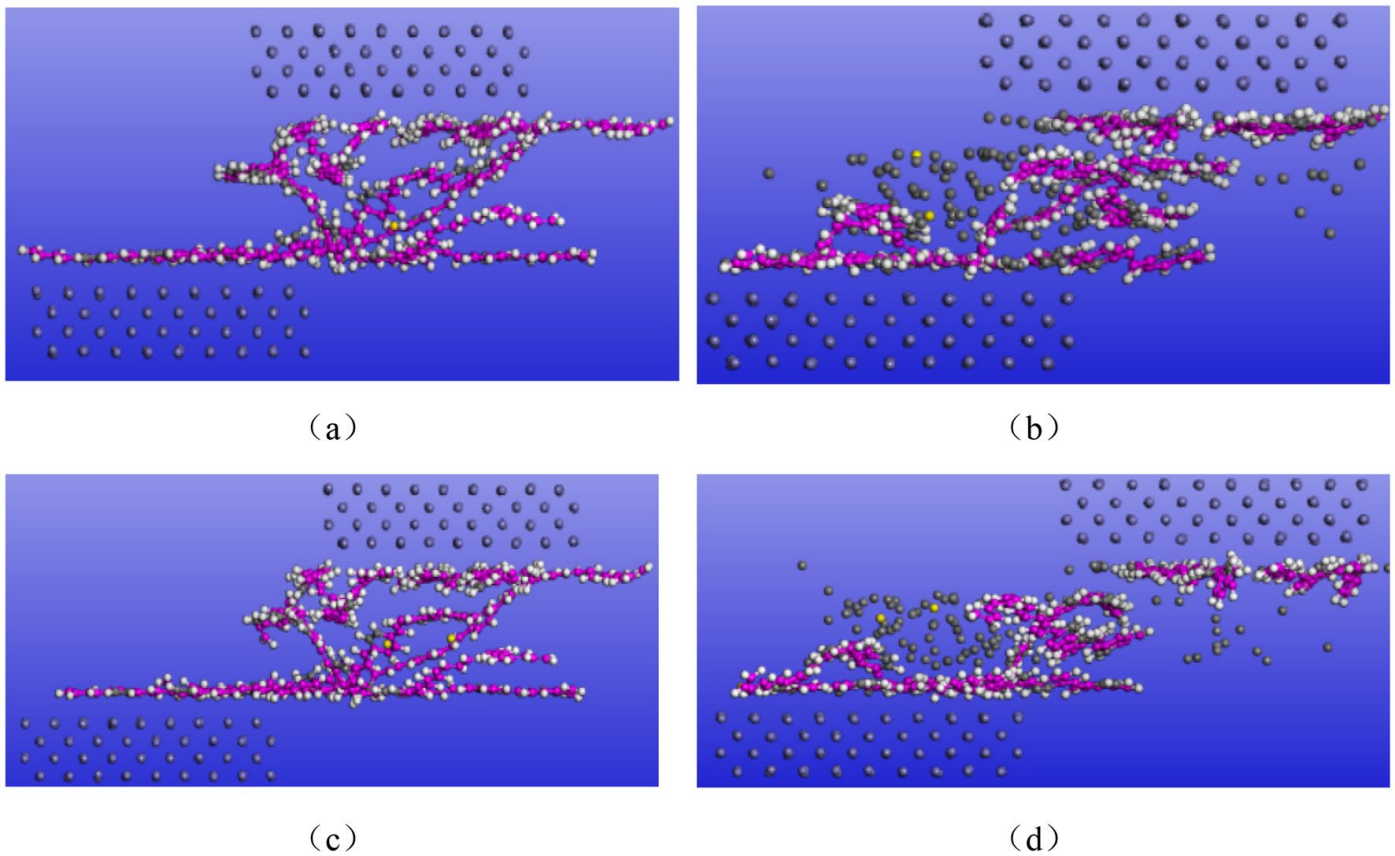
Schematic representation of the cut simulation: (**a**) the shear state of the pure SBR polymer at 1000 ps; (**b**) the shear state of the SBR polymer at 1000 ps after the addition of C atoms: (**c**) the simulated state when the pure SBR polymer is wholly cut and (**d**) the simulated state of the SBR polymer after the addition of C atoms.

The shear simulation process is shown in Fig. 7. Figure 7a shows the shear state of pure SBR polymer simulation at 1000 ps, and Fig. 7b shows the shear state of SBR polymer composite simulation at 1000 ps after adding a C atom. Figure 7c shows the simulated state of pure SBR polymer under complete shear and Fig. 7d shows the simulated state of the SBR polymer under complete shear after adding C atoms. It can be seen that the polymer chain of pure rubber polymer composite is deformed to a large extent under the shear action of the Fe layer.

In contrast, the polymer composites reinforced by adding C atoms are less deformed by the Fe layer, and the introduction of C atoms can effectively improve the shear deformation resistance of the polymer. In the actual application process, it can better resist the influence brought by the movement of Fe atoms, make the rubber matrix of the rubber conveyor belt and steel wire rope skeleton more stable, avoids the damage of rubber matrix caused by the small sliding of steel wire rope, and improve the reliability of the working process of steel wire rope rubber conveyor belt. As the working process of the steel wire rope rubber conveyor belt covers a wide range of temperatures, the simulation is carried out under the conditions of − 40 °C, − 30 °C, − 20 °C, − 10 °C, 0 °C, 10 °C, 20 °C, 30 °C and 40 °C respectively, although the simulation results are not the same, the velocity, temperature and relative atomic concentration of SBR polymer composite in the thickness direction are still similar to the temperature, kinetic energy, mechanical energy and pressure of the whole system during the simulation process after adding C atom reinforcement. Here, the shear simulation at 30 °C is taken as an example. In most cases, the rubber material equipment is used at room temperature. Due to some friction during the use process, the temperature of the rubber material is often around 30 °C. Therefore, 30 °C was selected for the shear simulation.

**Figure 8:**
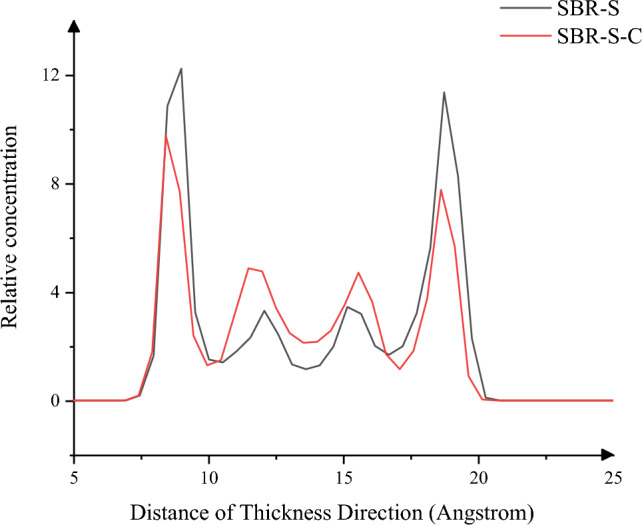
Thickness direction Relative concentration.

In order to further study the influence of adding C atoms on the shear process of SBR polymer composites, the “Forcite Analysis” function was used to analyze the concentration distribution in the thickness direction^26^. As shown in Fig. 8, the maximum relative atomic concentration in the thickness direction appears on both sides in contact with the Fe layer. After adding C atoms on both sides, the stability of both sides is improved, and the relative concentration is decreased. One side decreases from 12.25 to 9.76 (reduce 20.33%), and the other decreases from 11.37 to 7.78 (reduce 31.57%). The peak atomic concentration of SBR polymer composite reinforced by adding C atoms into the polymer matrix is lower than that of pure SBR polymer composites. SBR polymer composite tends to have adsorption between C atoms and SBR polymer composites. When the polymer matrix is subjected to shear load, the peak atomic concentration of SBR polymer composite is lower than that of pure SBR polymer composites. The rubber polymer composites atoms without C atoms are inclined to move to the friction region between the iron layer in the polymer matrix and the polymer matrix. The rubber polymer matrix binds to the C atoms instead of moving to the Fe layer, improving the stability of the shear process of the composite.

In Liu et al.^25^, nitrile butadiene rubber's mechanical and tribological properties were studied at a molecular scale by adding nano-SiO_2_, and the influence of the change of adding SiO2 temperature and velocity on the material properties was discussed. It was concluded that the polymer properties were improved by reducing the peak velocity and peak temperature after adding SiO_2_.

**Figure 9:**
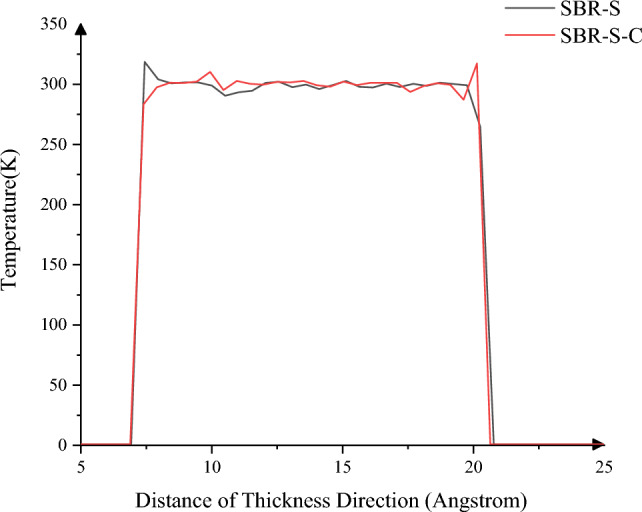
Temperature change in the thickness direction.

In addition, the temperature change along the thickness direction is also studied. As shown in Fig. 9, the local maximum temperature of 318.54 K decreases to 317.77 K. The SBR polymer composites, without adding C atoms, have a violent internal interaction during shear, including many bond breaking and formations. Hence, the stability of SBR polymer composites is poor. At the same time, high temperature will lead to a large amount of energy dissipation, resulting in the heating of SBR polymer composite material; the increase in temperature will lead to more vital molecular activity so that the reliability of the material is reduced again. On the macro level, due to the increase in temperature, the rubber will produce an aging phenomenon, leading to the shortening of the service life of the rubber. In actual work, heating will lead to the aging of the rubber matrix of the conveyor belt, which can easily cause damage and affect the service life. Introducing C atoms for reinforcement can effectively reduce the peak temperature and improve the stability of SBR polymer composite material.

**Figure 10:**
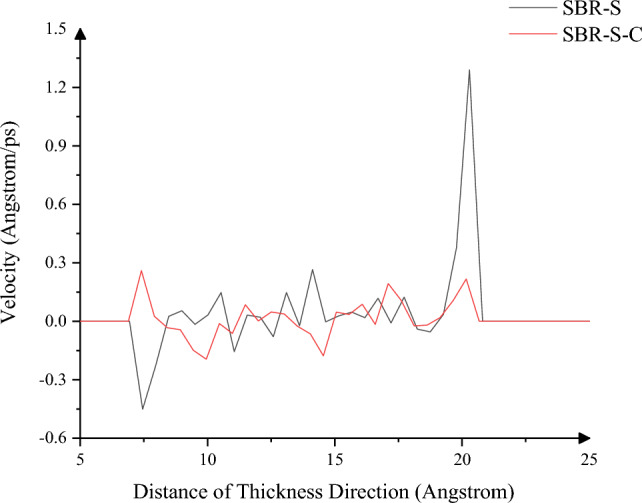
Variation of the velocity in the thickness direction.

The profile velocity in the thickness direction was studied, as shown in Fig. 10. The average and maximum velocity before C atoms reinforcement were 0.15 Angstrom/ps and 1.29 Angstrom/ps, respectively. After C atoms reinforcement, the average speed and maximum speed are 0.08 Angstrom/ps and 0.26 Angstrom/ps, decreased by 46.67% and 79.84%, respectively. The overall average velocity is higher as the temperature and atomic motion of SBR polymer composites reinforced with no C atom are higher in the shear process. At the same time, the mutual adsorption between SBR polymer composites and Fe atoms before C atoms reinforcement is more significant than after C atoms reinforcement. There is adhesion near the Fe atoms at both ends, which tends to follow the motion of the Fe layer so that it will have a higher movement speed. In addition, because the internal interaction of SBR polymer composites without adding C atoms is more intense in the shear process, along with the breaking and formation of chemical bonds, chemical reactions between atoms will occur, and the relative motion of atoms is inevitable. Due to the tendency of SBR polymer composite material to follow the movement of iron atoms on the contact surface with Fe atoms, the stability of the material is reduced, and it is easy to slide under shear. The rubber matrix of a conveyor belt without C atoms reinforcement is more prone to tear, which can be weakened after C atoms reinforcement to improve the reliability of the working process of the rubber conveyor belt.

**Figure 11:**
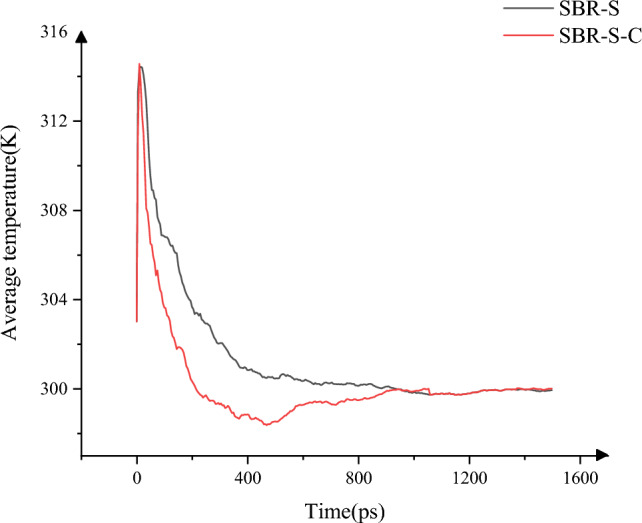
System average temperature change.

The change in the overall average temperature of the system was studied^27^. As shown in Fig. 11, the maximum average temperature of the system decreased from 314.56 to 314.42 K, and the average temperature decreased from 301.43 to 300.21 K. The maximum temperature of the system occurred between 5 and 15 ps, at which time the shear motion began. The SBR polymer composite material slides relatively under the action of Fe atoms slip, resulting in the interaction between atoms generating heat, resulting in the system temperature rise. Adding C atoms can significantly reduce the system's average temperature. The system's maximum average temperature and the temperature of the whole shearing process are significantly improved after the addition of C atoms. Reducing the temperature from the microscopic point of view can weaken the molecular movement and improve the application reliability of the conveyor belt. Reducing the temperature in actual work can reduce the aging of the rubber matrix of the conveyor belt, improve the reliability of the working process of the conveyor belt, and extend the service life of the conveyor belt.

**Figure 12:**
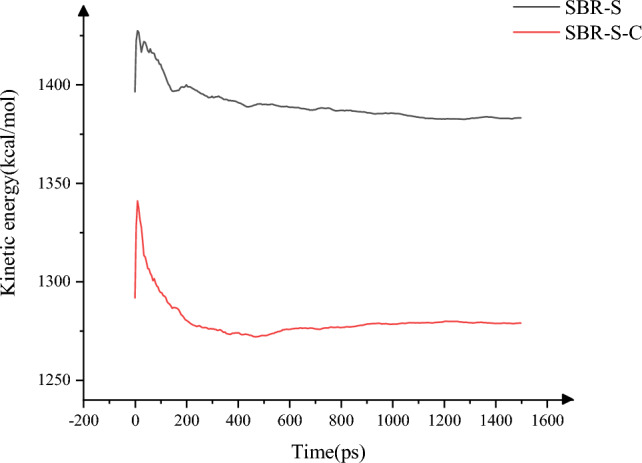
Kinetic energy.

The velocity of the system is closely related to the kinetic energy of the system. The velocity has been studied above. In order to ensure the comprehensiveness of the study, the kinetic energy before and after adding C atoms to the system is studied here. In the study of the shear process, the kinetic energy of SBR polymer composite material changes, as shown in Fig. 12. The maximum kinetic energy of the system decreases from 1427.38 to 1340.84 kcal/mol, and the average kinetic energy decreases from 1389.92 to 1279.91 kcal/mol. 2.62% and 4.54%, respectively. The movement of molecules generates kinetic energy, and when adding C atoms, the system's kinetic energy decreases. When carbon atoms are added, polymer molecules interact with carbon atoms, reducing the interaction between polymer molecules and iron atoms. In the shearing process of the upper and lower layers of Fe atoms, the polymer weakens along with the movement trend of Fe atoms. The kinetic energy of the polymer decreases after the addition of C atoms in the shearing process, and the stability of the polymer is enhanced. Therefore, the stability of the SBR polymer composites can be effectively improved by weakening the polymer failure under shear.

**Figure 13:**
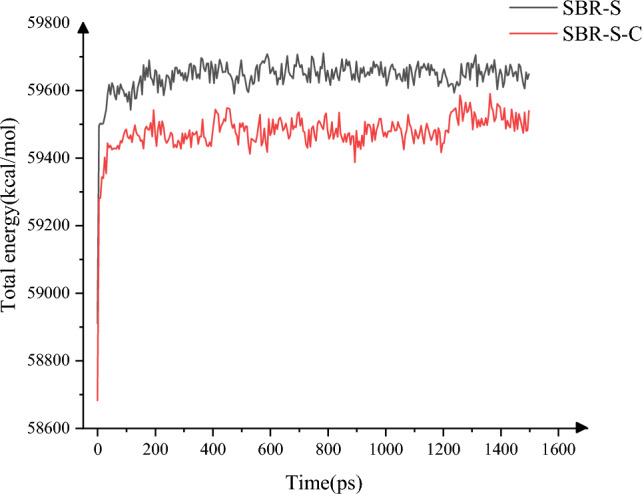
Total energy variation.

The Hamiltonian method is adopted to calculate the system's total energy in the shear simulation, as shown in Fig. 13. The average and maximum of the system's total energy changed from 59,640.61 kcol/mol and 59,710.38 kcol/mol to 59,477.34 kcol/mol and 59,589.98 kcol/mol, respectively. Adding C atoms can effectively reduce the energy of SBR polymer composites. The polymer composite is more stable in the shearing process of Fe atoms and is not easy to move with Fe atoms and react to itself, which improves the stability of SBR polymer composites. When applied to the rubber matrix of the conveyor belt, the wear brought by the friction between the belt framework and the rubber matrix can be reduced^27^.

**Figure 14:**
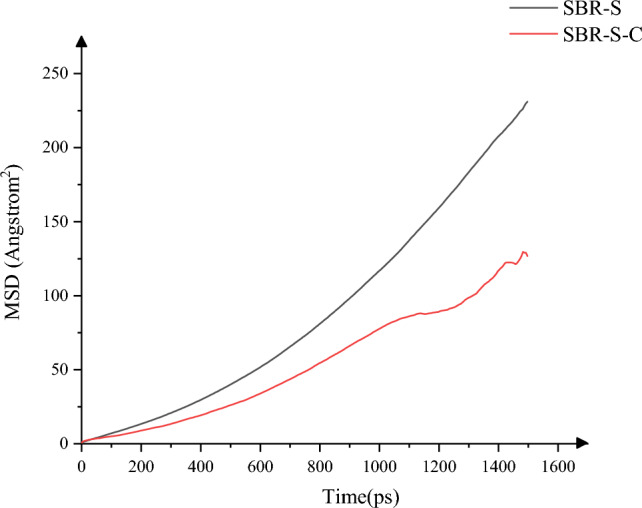
MSD change.

The change of mean square displacement (MSD) of SBR polymer composites in the shear process of Fe atoms is studied^28^, as shown in Fig. 14, the relationship between MSD of polymer composites and time indicates that the flow of polymer composites is restricted to a certain extent after the addition of C atoms, and the maximum and average values of MSD before the addition of C atoms are 230.85 and 87.81, respectively. After adding C atoms, the maximum and average values of MSD are 129.32 and 52.95, which decrease by 43.98% and 39.70%, respectively. The addition of C atoms can effectively limit the flow of polymer composite material and improve the stability of polymer composite material. When applied to the rubber conveyor belt, the wear caused by the interaction between the steel wire rope and the rubber matrix can be weakened, and the reliability of the working process of the steel wire rope rubber conveyor belt can be improved. By studying the interaction of Fe atoms with SBR polymer, we found that the SBR polymer performs better by adding C atoms. Can deduce: extend to the wire rope rubber conveyor belt between the role of wire rope core and rubber is the Fe atoms and the role of SBR polymer, SBR after adding C atoms SBR rubber, then the SBR rubber material wire rope core rubber conveyor belt after adding C atoms strength will be improved, improve reliability, prolong the service life of rubber conveyor belt, reduce the production cost.

In conclusion, adding C atoms to the SBR polymer composites can improve the stability of the SBR polymer composites. The relative atomic concentration, velocity, and temperature of the upper and lower layers of Fe atoms on the thickness direction of the intermediate layer of SBR polymer composites are reduced during the shear process. The value fluctuation is weakened during the shear process, and the stability is enhanced. During the shear simulation, the average temperature, kinetic energy, and total energy of the system were all reduced, and the stability of the SBR polymer composites was improved as a whole during the shear simulation. After adding C atoms to the simulation, the change of pressure was weakened, indicating that the self-reaction of the SBR polymer composites was weakened, and the stability of the polymer composites was enhanced during the shear process. When SBR polymer composite material is applied to steel wire rope rubber conveyor belts, the influence brought by the interaction between steel wire rope skeleton and rubber matrix is weakened, the working process of the conveyor belt is more reliable, and the service life is extended.

### Non-isothermal conditions

Friction will occur between Fe atoms and SBR polymer composites during the shearing process, and a large amount of energy will be dissipated when friction occurs. According to Fleisher's theory (1973), only 9–16% of the energy generated during the friction process can be stored in the material, and most of it will be dissipated in the form of friction heat^25^. As the shearing process continues, friction continues to occur, energy will continue to be generated, more and more energy will accumulate in the material, and when the energy reaches a critical value, the material will break away from each other. The material will fail due to the accumulation of energy. Under the same pressure, the greater the friction coefficient of the material, the greater the friction between the materials, the more heat energy generated when the friction occurs, and the greater the possibility of material failure. Therefore, in each frame between the Upper and Lower SBR polymer composite and the Fe layer in the process of sampling shear simulation, the x-direction and Z-direction forces represent the friction force and the Z-direction is the pressure. Calculate the average of the X and Z forces, and further calculate the friction coefficient µ.

2 $$\mu = \frac{{F_{x} }}{{F_{z} }}$$

where *Fx* is the force in X direction on the surface of SBR polymer model [Gpa]; *Fz* is the force in Z direction on the surface of BR polymer model [Gpa]; µis the coefficient of sliding friction. The change of friction coefficient before and after adding C atom is shown in Table 1, in which the unit of force is [Gpa].

**Table 1:** The change of friction coefficient before and after adding C atom.

	Temperature/°C	Upper				Lower		
		*F*_*x*_	*F*_*z*_		*µ*	*F*_*x*_	*F*_*z*_	*µ*
SBR-S	− 40	0.04		0.41	0.10	0.05	0.42	0.12
SBR-S-C		0.06		1.25	0.05	0.04	1.30	0.03
SBR-S	− 30	0.06		0.50	0.12	0.07	0.53	0.13
SBR-S-C		0.04		1.22	0.03	0.03	1.19	0.03
SBR-S	− 20	0.04		0.39	0.10	0.05	0.37	0.14
SBR-S-C		0.05		1.29	0.04	0.05	1.27	0.04
SBR-S	− 10	0.06		0.40	0.15	0.05	0.39	0.13
SBR-S-C		0.03		1.36	0.02	0.03	1.35	0.02
SBR-S	0	0.04		0.24	0.17	0.05	0.23	0.22
SBR-S-C		0.07		1.28	0.05	0.07	1.28	0.05
SBR-S	10	0.04		0.38	0.11	0.04	0.39	0.10
SBR-S-C		0.05		1.33	0.04	0.05	1.33	0.04
SBR-S	20	0.03		0.36	0.08	0.03	0.31	0.10
SBR-S-C		0.03		1.28	0.02	0.03	1.36	0.02
SBR-S	30	0.04		0.50	0.08	0.04	0.51	0.08
SBR-S-C		0.03		1.36	0.02	0.07	1.35	0.05
SBR-S	40	0.02		0.21	0.10	0.02	0.23	0.09
SBR-S-C		0.05		1.37	0.04	0.03	1.43	0.02

**Figure 15:**
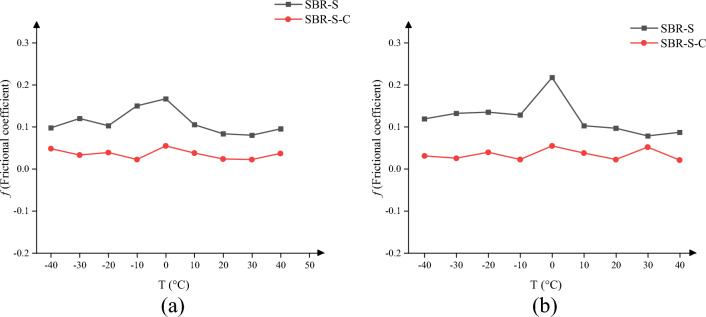
Change of friction coefficient: (**a**) Upper frictional coefficient; (**b**) Lower frictional coefficient.

Based on the data in Table 1, the change of friction coefficient as shown in Fig. 15 is obtained. No matter the current working temperature, the friction coefficient of SBR polymer composite is significantly reduced after the addition of C atom. Due to the wide application temperature range of SBR polymer composite, the binding energy is relatively stable in the range of − 40 to 40 °C. At − 40 °C, − 30 °C, − 20 °C, − 10 °C, 0 °C, 10 °C, 20 °C, 30 °C, 0.15 °C, 0.17, 0.11, 0.08, 0.08 and 0.10 respectively, the Upper friction coefficient decreases from 0.10, 0.12, 0.10, 0.10, 0.10 to 0.05, 0.03, 0.04 and 0.02, 0.05, 0.04, 0.02, 0.02, 0.04, with an average decrease of 0.07, The Lower friction coefficient was reduced from 0.12, 0.13, 0.14, 0.13, 0.22, 0.10, 0.10, 0.08, 0.09 to 0.03, 0.03, 0.04, 0.02, 0.05, 0.04, 0.02, 0.05, 0.02, respectively, with an average decrease of 0.08. The reduction of friction coefficient can effectively reduce the heat generated by the friction between Fe atoms and SBR polymer composites, reduce the accumulation of energy, and prevent material failure due to excessive energy accumulation.

The binding energy between the SBR polymer composite and the Fe layer also affects the reliability of the material, According to Formula (3), the binding energy between the SBR polymer matrix and Fe atoms is calculated as follows:

3 $$U_{cohesive\;energy} = U_{total} - U_{polymer} - U_{Fe}$$

$U_{total}$ is the total potential [kcal/mol] of the three-layer structure; $U_{polymer}$ is the total potential [kcal/mol] of the SBR polymer composite; $U_{Fe}$ is the total potential [kcal/mol] of the Fe layer; $U_{cohesive} \;_{energy}$ is the binding energy [kcal/mol] between the SBR polymer composite matrix and the Fe surface. Based on the molecular dynamics simulation results, the energy of the SBR polymer composites before and after C-atomic enhancement at − 40 °C, − 20 °C, − 10 °C, 0 °C, 10 °C, 20 °C, 30 °C and 40 °C, was calculated as shown in Table 2 and all energy units in Table 2 are [kcal/mol].

**Table 2:** Energy before and after the addition of C atoms.

	Temperature/°C	$U_{total}$	$U_{polymer}$	$U_{Fe}$	$\left. {\left| {U_{cohesive\;energy} } \right.} \right|$
SBR-S	− 40	− 60,865.94	− 59,925.91	815.39	1755.42
SBR-S-C		− 61,024.88	− 59,956.85	667.94	1735.97
SBR-S	− 30	− 60,884.64	− 59,925.71	797.75	1756.68
SBR-S-C		− 61,003.45	− 59,917.15	648.60	1734.90
SBR-S	− 20	− 60,914.22	− 59,898.05	737.31	1753.48
SBR-S-C		− 60,867.80	− 59,921.57	786.84	1733.07
SBR-S	− 10	− 60,855.75	− 59,899.10	784.77	1741.42
SBR-S-C		− 60,887.34	− 59,900.33	739.39	1726.40
SBR-S	0	− 60,799.99	− 59,849.37	813.61	1764.23
SBR-S-C		− 60,825.69	− 59,862.99	783.53	1746.23
SBR-S	10	− 60,749.63	− 59,869.51	881.47	1761.59
SBR-S-C		− 60,824.97	− 59,861.38	778.07	1741.66
SBR-S	20	− 60,742.27	− 59,818.64	839.44	1763.07
SBR-S-C		− 60,859.86	− 59,878.34	761.47	1742.99
SBR-S	30	− 60,764.66	− 59,812.15	812.24	1764.75
SBR-S-C		− 60,827.01	− 59,862.86	777.67	1741.82
SBR-S	40	− 60,707.4	− 59,820.92	879.3	1765.78
SBR-S-C		− 60,742.54	− 59,825.91	826.98	1743.61

**Figure 16:**
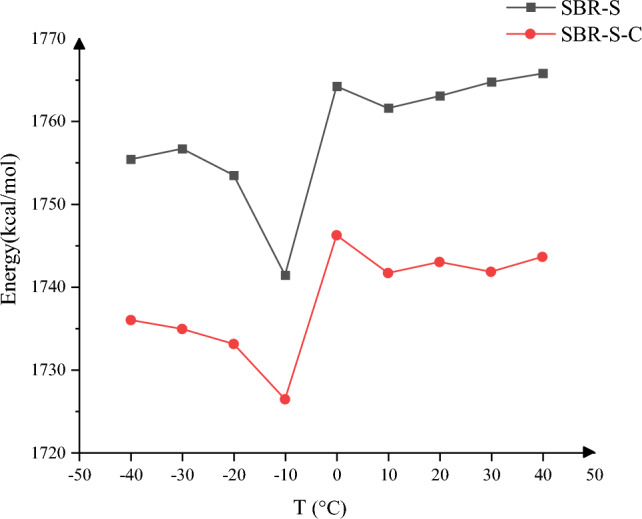
Cohesive energy change.

Based on the data in Table 2, the binding energies between SBR polymer composites with and without C atoms and Fe layers at different temperatures were processed, and the changes in binding energies at different temperatures were obtained, as shown in Fig. 16. No matter the working temperature of SBR polymer composites, the binding energy decreases significantly after adding C atoms. Because the application temperature range of SBR polymer composites is vast, the binding energy is relatively stable in the range of − 40 to 40 °C. At − 40 °C, − 30 °C, − 20 °C, − 10 °C, 0 °C, 10 °C, 10 °C, 20 °C, 30 °C, 30 °C, 40 °C, the binding energy decreased by 19.45, 21.78, 20.41, 15.02, 18.00, 19.93, 20.08, 22.93, 22.17, respectively, and the average decreased by 19.98. The greater the binding energy between Fe and SBR polymer, the stronger the interaction. When shear occurs, due to the strong interaction between Fe atoms and SBR polymer, the movement of SBR polymer with the movement of Fe atoms will lead to the large deformation of SBR polymer or even severe tear. Therefore, decreasing binding energy can weaken the interaction between the SBR polymer composites and the Fe layer. The adhesion between the Fe layer and the SBR polymer composites will be weakened during the shear process, reducing the deformation of the SBR polymer composites and improving their stability of the SBR polymer composites during the shear process.

**Figure 17:**
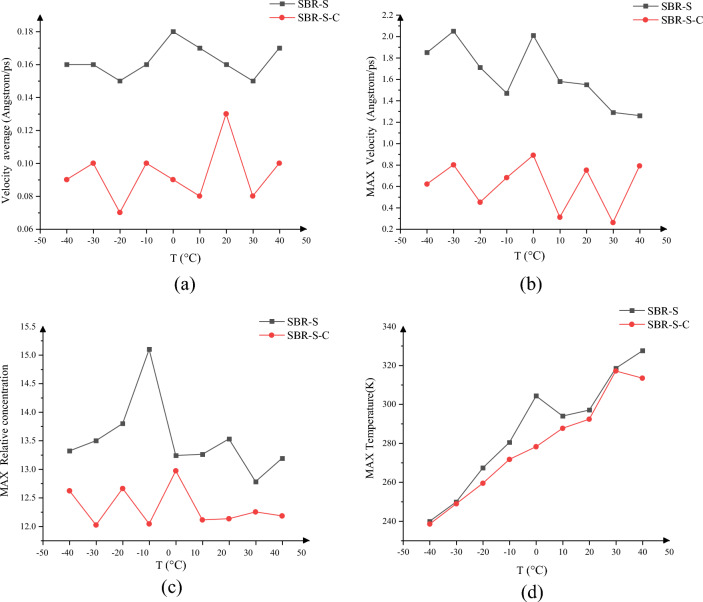
The thickness direction parameter change: (**a**) average velocity in the thickness direction; (**b**) maximum atomic velocity in the thickness direction; (**c**) maximum value of the atomic concentration; (**d**) maximum temperature in the thickness direction.

The maximum atomic velocity, average atomic velocity, maximum concentration, and maximum temperature in the thickness direction were studied as a function of ambient temperature, as shown in Fig. 17. Figure 17a shows the variation of the average velocity under different temperature conditions in the thickness direction. The decrease in the velocity variation range after adding a C atom can weaken the influence of temperature on SBR polymer composites. When the C atom is not added at − 40 to 40 °C, the maximum average velocity of 0.18 Angstrom/ps occurs at 0 °C. After adding a C atom, the maximum average velocity is 0.13 Angstrom/ps, which appears at 20 °C. Figure 17b shows the maximum atomic velocity in the thickness direction. The maximum atomic velocity decreases significantly after adding a C atom at any temperature. When the C atom is not added at − 40 to 40 °C, the maximum atomic velocity appears at − 30 °C, 2.05 Angstrom/ps, 0.89 Angstrom/ps; Fig. 17c shows the maximum atomic concentration under various temperature conditions. The atomic concentration after adding C atoms is lower than before. Without adding C atoms, the maximum atomic concentration appears at − 10 °C (15.1), and when adding C atoms, the maximum atomic concentration appears at 0 °C (12,97). Figure 17d shows the maximum temperature in the thickness direction. The stability is improved after the addition of C atoms, and the temperature increases with the increase of ambient temperature during the shearing process, which is significantly improved compared with the violent fluctuation and irregular change of temperature caused by the shearing action before the addition of C atoms. The temperature changes are relatively stable, and the maximum temperature before adding the C atom is 327.51 K. It appears at 40 °C, and the maximum temperature after adding C atoms appears at 30 °C, which is 317.19 K.

**Figure 18:**
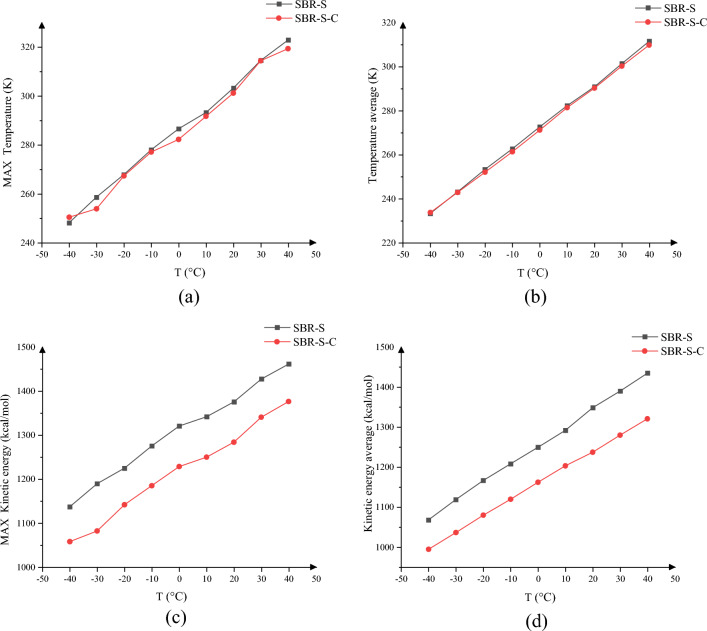
System temperature and kinetic energy change: (**a**) the maximum temperature of the system simulation process; (**b**) the overall average temperature of the shear simulation; (**c**) the maximum kinetic energy of the shear simulation process and (**d**) the overall average kinetic energy of the system.

The temperature and kinetic energy of the whole SBR polymer composites in the simulation process are closely related to the stability of the polymer. Therefore, the average temperature and kinetic energy of the whole polymer in the whole shear process before and after adding C atoms, as well as the maximum temperature and kinetic energy of the system in the whole shear process, were analyzed, and the overall temperature and kinetic energy of − 40 to 40 °C were obtained, as shown in Fig. 18.

Figure 18a shows the maximum temperature of the simulated process of the system under different ambient temperature conditions, Fig. 18b shows the overall average temperature of the system during the shear simulation at different ambient temperatures, Fig. 18c shows the maximum kinetic energy of the system shear simulation process at different ambient temperature, Fig. 18d shows the overall average kinetic energy of the system during the simulation, The kinetic energy and temperature of the SBR polymer composite were reduced after the overall addition of the C atomic enhancement, Lower temperature is able to reduce the molecular movement, Can also reduce the material aging due to heating, The reduced kinetic energy characterized the weakened molecular motion, The stability of the SBR polymer composite is enhanced when the molecular motion is weakened, The stability of all the SBR polymer composites was improved, Applied to the wire rope rubber conveyor belt, Ability to reduce the effect due to the interaction between the wire rope and the rubber, Improve the reliability of the conveyor belt work.

**Figure 19:**
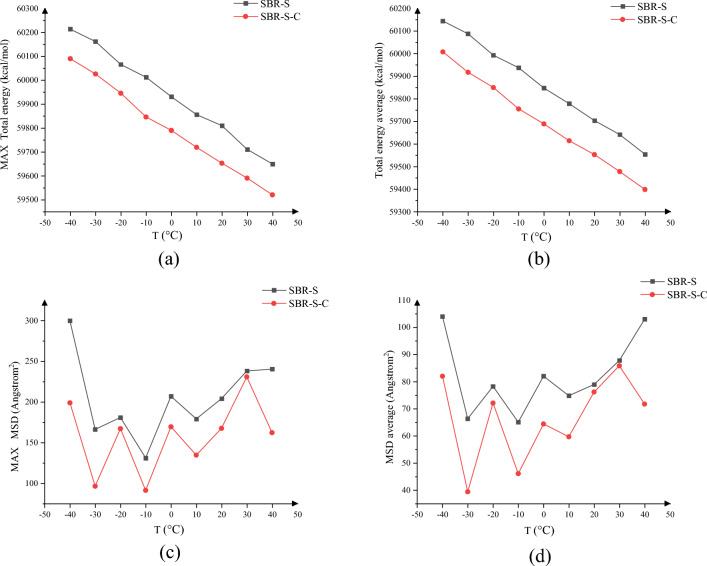
Changes in system energy and MSD: (**a**) maximum total energy of SBR polymer composites; (**b**) average total energy of SBR polymer composite; (**c**) maximum MSD of SBR polymer composite; (**d**) average MSD of SBR polymer composite.

The total energy and MSD of polymer composites can effectively characterize the current reaction. Therefore, the total energy and pressure at different temperatures are studied, as shown in Fig. 19. Figure 19a shows the maximum value of total energy of SBR polymer composites under different ambient temperature conditions, Fig. 19b shows the average value of total energy of SBR polymer composites under different ambient temperature conditions, Fig. 19c shows the maximum value of SBR polymer composite under different ambient temperature conditions, and Fig. 19d means the MSD of SBRs polymer composites under different ambient temperature conditions. After the enhancement by adding the C atom, At ambient temperatures of − 40 °C, − 30 °C, − 20 °C, − 10 °C, 0 °C, 10 °C, 20 °C, 30 °C, and 40 °C. The mean reduction of the total energy of the SBR polymer composites were 136.30, 170.30, 143.20, 182.30, 159.01, 164.30, 150.80, 164.30, 156.20, respectively, All achieve a reduction, Average decrease of 158.52; The maximum reduction of the total energy of the SBR polymer composites were 123.50, 135.70, 120.20, 166.50, 140.93, 136.50, 157.20, 120.40,1 29.60, respectively. Average decrease of 136.73; The maximum values of the SBR polymer composite MSD decreased by 33.66%, 42.09%, 7.61%, 30.39%, 18.15%, 24.84%, 17.88%, 3.02%, 32.52%, respectively, Lower significantly, The average decline was 23.35%. The mean values of MSD of SBR polymer composites decreased by 21.15%, 40.72%, 7.86%, 29.22%, 21.51%, 20.26%, 3.50%, 2.21%, 30.40%, respectively. The average drop was 19.65 percent. The addition of C atoms reduces the energy maximum with the average value, Improving the stability of SBR polymer composites during shear processes, Reducing the effect of the shear action on the composites, Improve the stability of composite materials; MSD of the composite shear process by adding C atoms, Making the molecules in the composite to shear by the Fe atoms, The weakened movement of the molecules itself, To optimize the performance, Improve the stability of the polymer composites, The interaction of conveyor belt rubber matrix and wire rope core is weakened during the practical application, When the deformation is reduced by the same shear action, Increasing improvement in the reliability of the conveyor belt.

## Conclusion

In this paper, Material Studio software is used to complete the modeling and dynamic simulation of SBR polymer composite materials, and the SBR polymer composite material model and the three-layer model of Fe-SBR polymer composite material are established. The upper and lower layers of Fe atoms exert shear loads on SBR polymer composite materials during the dynamic simulation process. By comparing before and after the introduction of C atom, the influence of the introduction of C atom on the elastic modulus and material stability of SBR polymer composite was studied. The elastic modulus of the material was analyzed based on the simulation results of SBR polymer composite model at 30 °C. It is found that the maximum and average values of the X-direction tensile elastic modulus before adding C atom are 4.89 Gpa and 0.58 Gpa, respectively, and the maximum and average values of the tensile elastic modulus after adding C atom are 8.41 Gpa and 1.35 Gpa, respectively, increasing by 72.02% and 132.43%. The maximum elastic modulus in the Y direction increased from 5.90 to 8.12 Gpa, an increase of 37.67%; the average value increased from 0.69 to 1.61 Gpa, an increase of 132.94%; the maximum elastic modulus in the Z direction increased from 5.27 to 8.67 Gpa, an increase of 64.31%. The average elastic modulus is increased from 1.11 to 1.34 Gpa by 21.07%. In terms of compressive elastic modulus, the maximum elastic modulus in the X direction increases from 1.68 to 5.85 Gpa, an increase of 248.33%, and the average elastic modulus increases from 0.60 to 2.15 Gpa, an increase of 255.81%. The maximum elastic modulus in the Y direction was increased from 2.40 to 6.22 Gpa by 158.65%, and the average elastic modulus was increased from 0.72 to 1.71 Gpa by 137.64%. The maximum elastic modulus in the Z direction increased from 2.18 to 5.97 Gpa by 174.24%, and the average elastic modulus increased from 0.47 to 2.65 Gpa by 458.89%. In terms of shear modulus, the maximum elastic modulus of XY plane increases from 2.15 to 5.56 Gpa, an increase of 159.28%, and the average elastic modulus increases from 0.23 to 0.58 Gpa, an increase of 152.53%. The maximum elastic modulus of the XZ plane is increased from 2.22 to 5.93 Gpa, an increase of 167.57%, and the average elastic modulus is increased from 0.22 to 0.52 Gpa, an increase of 130.44%. The maximum elastic modulus of YZ plane is increased from 1.61 to 4.48 Gpa by 177.61%, and the average elastic modulus is increased from 0.20 to 0.59 Gpa by 193.03%. The stability of the material was analyzed by using a three-layer model at 30℃. The average and maximum velocities before C atom was added were 0.15 Angstrom/ps and 1.29 Angstrom/ps, respectively. After the addition of C atom, the average and maximum velocities were 0.08 Angstrom/ps and 0.26 Angstrom/ps, which decreased by 46.67% and 79.84%, respectively, and the movement of atoms with Fe layer was weakened. The maximum atomic concentration decreased from 12.25 to 9.76, a decrease of 20.33%; The other side decreased from 11.37 to 7.78, a decrease of 31.57%; Local maximum temperature 318.54 K decreased to 317.77 K; The maximum average temperature of the system decreases from 314.56 to 314.42 K, and the maximum temperature decreases from 301.43 to 300.21 K. The average and maximum values of total energy of the system vary from 59,640.61 kcol/mol and 59,710.38 kcol/mol to 59,477.34 kcol/mol and 59,589.98 kcol/mol, respectively. The maximum kinetic energy of the system decreased from 1427.38 to 1340.84 kcol/mol, and the average energy decreased from 1389.92 to 1279.91 kcol/mol, respectively, by 2.62% and 4.54%. The maximum value and average value of MSD before adding C atom were 230.85 and 87.81, respectively, and the maximum value and average value of MSD after adding C atom were 129.32 and 52.95, which decreased by 43.98% and 39.70%, respectively, with significant changes.

The dynamic simulation was studied before and after adding C atoms at ambient temperature changes between − 40 and 40 °C. The binding energy and frictional coefficient between SBR polymer composites and Fe atoms was found to decrease at any temperature between − 40 and 40 °C, the Upper and Lower friction coefficients decreased by 0.07 and 0.08, respectively, with binding energy an average decrease of 19.98 kcal/mol. The maximum velocity, average velocity, relative atomic concentration, and maximum temperature in the direction of thickness decreased, and the maximum velocity, average velocity, relative atomic concentration, and maximum temperature were weakened by the ambient temperature after the addition of C atoms, which improved the stability of SBR polymer composite. In addition, the average temperature, maximum temperature, average kinetic energy, and maximum kinetic energy of the whole SBR polymer composite also decreased after adding C atoms. The maximum total energy of SBR polymer composites, the average total energy of SBR polymer composites, the maximum pressure, and the average pressure of SBR polymer composites decreased significantly after adding C atoms. The maximum total energy of SBR polymer composites decreases by 136.73 kcal/mol on average, the average total energy of SBR polymer composites decreases by 158.52 kcal/mol on average, and the maximum MSD at each temperature decreases by 23.35% on average. The mean MSD at all temperatures decreased by 19.65%. In the shear simulation process, the interaction between SBR polymer composites and Fe atoms is weakened, and the stability is enhanced. In the actual application process, the rubber polymer matrix tends to bond with C atoms instead of moving to the Fe layer, thus improving the composite material's stability and the conveyor belt's reliability. At present, the vulcanizing machine has been purchased, and the raw materials and various fillers of the rubber matrix have begun to be purchased, and experimental research is planned. The enhancement of filler on the working performance of rubber materials and the influence of different fillers on the working performance of materials were discussed.

### Supplementary Information

Supplementary Information.

## Data Availability

The authors confirm that the data supporting the findings of this study are available within the article and its supplementary materials. The “Supplementary materials” Raw data records all the data and pictures involved in this article, xsgd-40 ~ xsgd40 and JQ30 record all the analyses in this article. All analyzed data are recorded in Data set in “Related files”. If other data is required, corresponding authors will supply the relevant data in response to reasonable requests.
